# Virtual bargaining: a theory of social decision-making

**DOI:** 10.1098/rstb.2013.0487

**Published:** 2014-11-05

**Authors:** Jennifer B. Misyak, Nick Chater

**Affiliations:** Behavioural Science Group, Warwick Business School, University of Warwick, Coventry CV4 7AL, UK

**Keywords:** virtual bargaining, social decision-making, strategic interaction, coordination

## Abstract

An essential element of goal-directed decision-making in social contexts is that agents' actions may be mutually interdependent. However, the most well-developed approaches to such strategic interactions, based on the Nash equilibrium concept in game theory, are sometimes too broad and at other times ‘overlook’ good solutions to fundamental social dilemmas and coordination problems. The authors propose a new theory of social decision-making—virtual bargaining—in which individuals decide among a set of moves on the basis of what they would agree to do if they could openly bargain. The core principles of a formal account are outlined (vis-à-vis the notions of ‘feasible agreement’ and explicit negotiation) and further illustrated with the introduction of a new game, dubbed the ‘Boobytrap game’ (a modification on the canonical Prisoner's Dilemma paradigm). In the first empirical data of how individuals play the Boobytrap game, participants' experimental choices accord well with a virtual bargaining perspective, but do not match predictions from a standard Nash account. Alternative frameworks are discussed, with specific empirical tests between these and virtual bargaining identified as future research directions. Lastly, it is proposed that virtual bargaining underpins a vast range of human activities, from social decision-making to joint action and communication.

## 1. Introduction

A crucial challenge for the theory of goal-directed decision-making in social contexts is that the results of an agent's actions may depend on the actions of other agents. Where this is so, it appears that, to determine whether any particular action will successfully achieve an agent's goals depends on inferring what the other agents will do. Yet a moment's reflection reveals that inferring the likely actions of other agents appears problematic. Any of the agents may reasonably wonder: ‘How can the other agents possibly infer what I'm going to do, when I don't yet know myself?’ There seems to be the danger that each agent may be ‘lost in thought’ forever, attempting to puzzle out what the others will do.

One way out of this puzzle is to abandon the attempt to think in terms of goals at all. So, for example, it is widely assumed that many aspects of group behaviour in animals emerge from the application of algorithmic rules, partially determining the action of a given animal, based on the actions of nearby animals. For instance, a variety of models of flocking show that a coherently moving flock may emerge from very simple local rules for adjusting the flight of each bird in the light of the movements of neighbouring birds [1–3]; and many aspects of human and non-human animal behaviour have been modelled as arises from herding, a tendency to copy the behaviour of neighbours (causing, for example, stampedes, and perhaps, stock market runs and riots) [4,5].

But if our focus is the theory of goal-directed behaviour, then a different approach is required. We need to face, squarely, the question of how several agents may attempt to pursue their own goals, in the knowledge that the consequences of their actions may be mutually interdependent. By far the most well-developed approach to this problem stems from the notion of Nash equilibrium and best-reply reasoning, developed in game theory.

**Figure 1.**
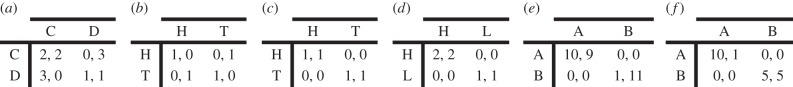
Various games represented in normal form matrices. The ‘Row’ player decides between strategies delineated by rows and the ‘Column’ player decides between strategies delineated by columns. Ordered values in cells represent pay-offs to Row and Column players, respectively, for each possible interdependent outcome. (*a*) PD game, with strategies of C (Cooperate) and D (Defect). (*b*) Matching Pennies game, with strategies H (Heads) and T (Tails). Row ‘wins’ if both players choose identically (i.e. Heads–Heads or Tails–Tails); Column ‘wins’ otherwise. (*c*) Simple coordination game, where players obtain a pay-off if they make the same move. (*d*) Hi–Lo coordination game. The equilibrium (H,H) yields higher pay-offs for both players than another pure strategy equilibrium of (L,L) (or even a mixed strategy equilibrium in which both players choose L with probability 2/3). (*e*) Battle-of-the-Sexes game. Players must coordinate between one equilibrium that is preferable for the Row player and another that is preferable for the Column player. (The game's name comes from an imagined couple, one of whom prefers, say, ballet, whereas the other prefers football. They must independently decide which event to attend; both will be utterly miserable if they do not make the same choice.) Here, (A,A) is fairly good for both players, whereas (B,B) is very bad for the Row player. (*f*) Coordination game, unsolvable by maximizing summed pay-offs. Intuition suggests that players will have common knowledge that, if faced with a choice agreeing to the pay-offs (10, 1) and (5, 5), the latter will prevail. (Both players know that the Column player will never agree to such an outrageously asymmetrical split.) Of course, if the players could redistribute resources after the bargain is complete, and can perfectly trust each other to do so, then they will always choose the equilibrium of the greatest summed pay-offs, to maximize the ‘spoils’ to be divided. We will not consider such post-bargain trading here.

In a Nash equilibrium, each agent makes a ‘best response’ to the action of the other agent—where ‘best’ is determined entirely by the agents' own pay-offs. For example, in a social interaction with the structure of the canonical Prisoner's Dilemma (PD; figure 1*a*) game, there is just one Nash equilibrium of (D,D) in which both players ‘Defect’, even though it would be better for both players to attain (C,C) by playing ‘Cooperate’. Mutual cooperation (C,C) is not a Nash equilibrium because either player can unilaterally switch from C to D and gain an improved pay-off (3 rather than 2). But (D,D) is a Nash equilibrium, because neither player can unilaterally improve their pay-off by switching (indeed, a player who unilaterally switches from D to C obtains 0 rather than 1).

So far we have considered ‘pure’ Nash equilibria, but many games also have ‘mixed strategy’ equilibria, where one or more players choose their actions probabilistically, rather than deterministically. For example, consider the Matching Pennies game (figure 1*b*). Each player must choose between Heads and Tails. One player wins if both choose identically; the other player wins if both choose differently from each other. No set of deterministic choices constitute a Nash equilibrium in this game, because the losing player is clearly not responding best to the other's move; indeed, the losing player can unilaterally switch to become the winner by changing their response. But Matching Pennies does have a mixed strategy Nash equilibrium: each player chooses Heads and Tails with 50% probability, literally by spinning a coin.

Nash [6,7] famously showed that for a very wide class of interactions between players, the ‘game’ had at least one Nash equilibrium (whether ‘pure’ or ‘mixed’). Indeed, many interesting games have several such equilibria. Consider, for example, a simple ‘coordination game’ (figure 1*c*) in which, unlike PD or Matching Pennies, the players' interests align, rather than conflict. Here, there are two ‘pure strategy’ equilibria of (H,H) and (T,T) (and, in fact, a further ‘mixed strategy’ equilibrium, where both players choose by spinning a fair coin).

In social interactions that can be modelled as a game with multiple equilibria, producing predictions about what players should choose requires *selecting* one equilibrium among others. The notion of a Nash equilibrium needs to be ‘refined’, to pick out one such equilibrium over the others.

The virtual bargaining account of social interaction that we propose and outline below provides one theory of how to select between equilibria. The idea, as we shall see, is that agents should prefer the equilibrium that they would select if able to openly bargain, by sending messages back and forth between them (even though we assume they do not *actually* send such messages—it is in this sense that the bargaining is ‘virtual’). To provide some initial motivation for this approach, consider another coordination game, Hi–Lo (figure 1*d*; [8]), where agreement on one option is clearly better than agreement on the other. According to the virtual bargaining perspective, the reason that H is clearly preferred to L is that it is obvious to both agents that, were they in a position to explicitly bargain with each other to determine what to do, they would immediately agree on (H,H).

We will consider how this can be modelled more formally below. But for now, let us provide some intuitions favouring the virtual bargaining approach. In particular, note that the account presumes that factors that would influence actual bargaining will tend to influence the virtual bargaining, which might involve the ‘mental simulation’ of an actual bargaining process. In real bargaining, for example, consider how one would react when faced with the two pure strategy equilibria in the ‘Battle-of-the-Sexes’ game (figure 1*e*). That is, imagine players who have to bargain concerning whether they should choose an option which has mutually good pay-offs (9 and 10 units) or one which has very asymmetrical pay-offs (12 and 1 units). Both players know that it is very unlikely they would mutually agree to choose the second, as the disadvantaged player will never concur. Thus, according to virtual bargaining, both players in this Battle-of-the-Sexes game will know that this is true, know that the other knows it, etc.; and hence both will spontaneously choose A, not B, alighting on the (A,A) equilibrium.

So far, one might wonder whether, rather than engaging in bargaining, both players are simply choosing the equilibrium of the greatest summed pay-offs. For example, rather than seeing the players as bargaining to achieve a solution, we might consider the players to reason instead as part of a ‘team’ whose objective is to maximize the total pay-off to the team members (see [8,9]). However, consider figure 1*f*, which provides an example where the summed pay-offs are so unfairly divided that they would not be agreed by real bargainers. The virtual bargaining account accordingly predicts that this equilibrium would not be selected.

The virtual bargaining account, as developed here, makes predictions based on the ‘goodness’ of virtual bargains, not how such bargains are arrived at. Thus, it is a type of equilibrium explanation, in the tradition of the Nash equilibrium and its refinements. But understanding the psychological processes that lead to a particular virtual bargaining outcome remains an important challenge. Indeed, a process-model may turn out to have important implications for the equilibria attained. For example, in games where people are rewarded when they independently choose the *same* item (e.g. the same place, time, colour; [10,11]), common mental processes may themselves be important clues concerning the ‘best’ item on which to virtually agree. Consider, for instance, two people who will receive a prize if they independently choose the same car brand. If *Ford* is the first brand of cars that comes to mind (presumably as a result of aspects of memory retrieval processes), then this may be a particularly natural suggestion for the ‘virtual agreement’ equilibrium [10]. Thus, combining processing factors with virtual bargaining may help explain why some equilibria serve as so-called ‘focal points’. We shall, however, set aside issues concerning multiple equilibria below.

## 2. Virtual bargaining solutions in social interactions

Thus far, it has appeared that the notion of a Nash equilibria is too broad. Interesting social interactions often have multiple Nash equilibria and require additional constraints for selecting one equilibrium above the others. Virtual bargaining provides one way to do this, among many others (e.g. trembling hand and subgame perfect Nash equilibrium [12], pay-off and risk dominance [13], ‘proper equilibria’ [14], Mertens-stable equilibrium [15], perfect Bayesian equilibrium [16] and sequential equilibria [17]).

But as a theory of goal-directed social interaction, the Nash equilibrium has a second drawback: many social interactions may not be Nash equilibria at all. There has been more than a half-century discussion of the fact that people frequently cooperate in PD [18–21]. This raises the possibility that people are, for example, concerned not only about their own pay-offs, but about the pay-off of the other player, and might be guided by feelings of altruism or norms of fairness, although there are also many other possible explanations [22–27].

Rather than add to this discussion here, though, we focus on a different source of additional possible ‘virtual bargains’, which can arise irrespective of whether participants in a social interaction have any altruistic feelings or sense of fairness.

**Figure 2.**
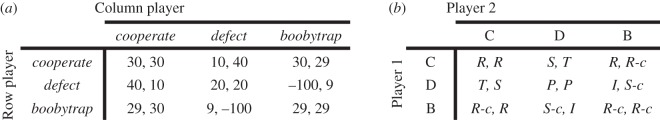
(*a*) A specific pay-off structure for the Boobytrap game. The ordinal relationships among values in the top-left inner quadrant of the matrix, comprising the outcome pay-offs for the strategy-subset *cooperate* and *defect*, are the same as those for PD (as illustrated previously in figure 1*a*). The Boobytrap game modifies a standard PD game by symmetrically adding the third strategy of *boobytrap*. (*b*) Generalized structure of the Boobytrap game. *R*, *S*, *T* and *P* are pay-off variables that follow standard inequalities of a PD game: *T* > *R* > *P* > *S*. If one player plays C (‘cooperate’) and the other plays D (‘defect’), then the cooperator obtains a low pay-off (*S*), and the defector a high pay-off (*T*). If both cooperate, they both receive a fairly good pay-off (*R*); but if both defect they receive the fairly bad pay-off (*P*). This is the standard structure in the well-known PD game. The ‘impact’ pay-off *I* occurs to a player who plays D when the other plays B (‘boobytrap’); *I* < *R*. The ‘cost’ (reduction in pay-off) of playing B is represented by *c*, which is positive-valued.

We illustrate this type of case by introducing a new game, which we call the ‘Boobytrap game’ (shown in figure 2*a*), and which we will explore experimentally below. The Boobytrap game is a modification of PD, in which an additional ‘move’ is added: the ‘boobytrap’. If a player chooses Boobytrap (B), their pay-offs will be precisely the same as if they had chosen Cooperate (C), except for a small reduction in each pay-off that can be conceptualized as the cost of ‘buying’ the boobytrap. Moreover, playing B is the same as playing C with respect to its effect on the *other* player's outcomes, with one crucial exception: if the other player chooses Defect (D), then that player receives a steep negative pay-off.

The Boobytrap game has just one Nash equilibrium of (D,D). The cost of ‘buying’ the boobytrap means that B yields a slightly lower pay-off than C, regardless of the action of the other player. Therefore, no pair of strategies involving B from either player can possibly be a Nash equilibrium, because switching from B to C will always be a better response to the other's move—whatever that move might be. By this logic, the players can infer that neither of them will choose B; and hence the game collapses back into a standard PD game, with the usual suboptimal (D,D) equilibrium.

But there is a better ‘bargain’ to be had: (B,B). Consider, for a moment, that the players can bargain face-to-face. They can immediately see that (B,B) is a better outcome than (D,D). Now (C,C) is better still, but the problem is that it is *unenforceable*. Assuming no altruism or similar, each player knows that, if they go through with their side of the bargain, the co-player may *exploit* them by Defecting—that is, the other player will be able to profit at their expense. But no such exploitation is possible for (B,B). Each player knows that *if* they go through with their side of the bargain, playing B, then no exploitation is possible; if the other plays D, then this will be mutually damaging. If the other plays B or C, then the first player obtains a good pay-off in either case.

So, there is a possible, non-exploitable bargain that has better pay-offs for both players than the Nash equilibrium. By playing B, a player deters the other from playing D. And hence the social interaction works to the benefit of both players. As both players know that this bargain is mutually advantageous, they can play it without having to explicitly bargain.

Here, then, the virtual bargaining account diverges sharply from most accounts which assume the Nash equilibrium as a starting point; in this game, a good virtual bargaining solution may not be a Nash equilibrium at all. We shall examine how people actually play the Boobytrap game presently.

The possibility of virtual bargaining between antagonists marks the division between the virtual bargaining account and an important set of related ideas developing the notion of ‘team-reasoning’ [8] and the related notion of ‘we-thinking’ [28,29]. Team reasoning allows the possibility for teams of people, not just individuals, to be viewed as agents—and that individuals who are members of a team may choose to behave in a way that fits their ‘role’ in that team. It is natural to think of teams as having common objectives (as in, for example, sports teams). Virtual bargaining can be viewed as providing a link between individual beliefs and values and the behaviour of the ‘team’—by viewing the preferences of the team as resulting from what ‘the team’ would agree, if its members had the opportunity to bargain. From this perspective, we can see the goals of the team as arising naturally from the goals of its members: the theory of virtual bargaining provides the crucial link.

## 3. A sketch of a formal theory of virtual bargaining

The theory of virtual bargaining can be made precise in a number of different ways. Here, we provide a sketch of the structure of such a theory, leaving technical developments and alternative analyses for future research. There are two core ideas. First, the notion of a *feasible agreement* picks out a set of possible ‘virtual bargains’, from which the players choose. The second step is the claim that, except in unusual circumstances which we shall not consider here, virtual bargaining is governed by the same principles that govern explicit negotiation.

The rudiments of a formal account can be encapsulated as follows, for the case where there are two agents. (A more general mathematical formulation is given in the electronic supplementary material.)

(i) A pair of strategies (whether ‘pure’, i.e. consisting of a single move; or ‘mixed’, consisting of a probability distribution over several moves) is *feasible*, if neither player can exploit the other to their own advantage. That is, given the strategy of the other, neither player can, by varying their strategy, gain advantage for themselves, to the disadvantage of the other.

Note that the set of feasible strategies is weaker than, and contains all, Nash equilibria. At a Nash equilibrium, neither player can unilaterally change their strategy to their own advantage *in any way at all*; this immediately implies, of course, that neither player can unilaterally change their strategy *to exploit the other* (i.e. so that the player benefits, to the disadvantage of the other).

How can the ‘goodness’ of a bargain be evaluated? In the absence of a well-developed formal theory of explicit negotiation (and perhaps with the expectation that such theory may not be possible), we should similarly not expect a complete formal theory of virtual bargaining. Nonetheless, we shall see that existing formal accounts of explicit bargaining, such as Nash's theory of bargaining [30], while incomplete, are nonetheless useful as a starting point for the analysis of virtual bargaining. Hence, we shall provisionally use

(ii) The goodness of a feasible bargain is, following Nash's theory of bargaining, the product of the utility gains to each player (relative to a no-agreement baseline) of adhering to that agreement (Nash showed that this particular goodness measure follows from very simple and natural axioms concerning bargaining).

Let us illustrate these steps by considering PD and then its modification, the Boobytrap game. For conventional PD, consider cases in which both players agree to adopt strategies with a non-zero probability of C. Then each player can exploit the other by not going through with their side of the bargain, but by playing D with probability 1. So no such pairs of strategies are ‘feasible’. Conversely, all strategies in which one player plays D with probability 1 *are* feasible, including the pure strategies (C,D), (D,C) and, of course, the Nash equilibrium (D,D).

Note that, by choosing D, each player can guarantee a pay-off of 20 units. But any bargain in which one player chooses D, but the other has a non-zero probability of playing C, will have a *negative* expected pay-off for the second player compared with the ‘baseline’ of also playing D. So the *product* of utility gains of the pair of players for any such bargain will be *negative*. The bargain (D,D) will have a 0 gain over this default option (because (D,D) *is* the default option for each player). So the ‘best’ feasible option is (D,D). So bargaining does not lead to an improved outcome: the theory of virtual bargaining does not, directly at least, help explain why people so frequently cooperate in one-shot PD.

Now let us consider the Boobytrap game. The game also has a single (D,D) equilibrium, which we shall take as the default outcome, where no bargaining occurs. In the specific Boobytrap game in figure 2*a*, the pay-offs are (20,20). Can bargaining lead to a better outcome? It turns out that this is possible. Suppose each player ‘buys’ a Boobytrap for certain. The pay-offs are now (29,29), and this is a feasible bargain, as neither player can exploit the other. To be sure, if one player knows that the other will play Boobytrap, then the first player can simply cooperate, thereby saving the ‘cost’ of the Boobytrap (and obtaining a pay-off of 30, rather than 29). But this leads to no disbenefit to the other player, who obtains pay-off 29 nonetheless. So (B,B) is a feasible bargain (albeit not a Nash equilibrium), and it is a better bargain for both players than that achieved by the Nash equilibrium.

Indeed, the players can do even better. They just need to buy the Boobytrap sufficiently often that the other player has no incentive to Defect. It turns out that the best possible bargain for each player in the specific boobytrap game we are considering is achieved when each player plays B with probability (infinitesimally above) 1/14; and otherwise plays C. This figure is obtained simply by considering the fraction of Bs required of a B/C mixture such that the co-player's pay-off by playing D (i.e. 40*p −* 100(1 *− p*), where *p* is the probability of playing C) is less than from playing C (i.e. 30), so that there is no incentive to play D. Simple algebra reveals that this holds when *p* < 13/14, and hence the probability of playing B is at least 1/14. Under this solution, expected pay-offs for each player are very nearly 30, and far better than the (20,20) pay-offs obtained in the Nash equilibrium.

Virtual bargaining predicts, then, that the players can find a bargain in which neither can exploit the other, but which is significantly better than the Nash equilibrium. Recall, though, that while intuitively natural, no solution involving playing B is predicted on standard game-theoretic analysis. While the Boobytrap is a potential deterrent against the co-player choosing D, it is a non-credible deterrent under Nash reasoning, because whatever the other player does, each player will always do better choosing C over B. The crucial empirical question is, therefore: how do people behave when confronted with the Boobytrap game?

## 4. Preliminary experimental test of virtual bargaining

We report below the first empirical data on how people play the Boobytrap game.

### (a) Methods (participants, materials and procedure)

Eighteen undergraduate and postgraduate students (13 women; age: *M* = 21.4, s.d. = 2.1) were recruited from the University of Warwick through an online participant recruitment panel. Participants were paid £5, in addition to any earnings (potential range from £0 to £7) determined proportionally from their pay-offs in one randomly selected game.

**Figure 3.**
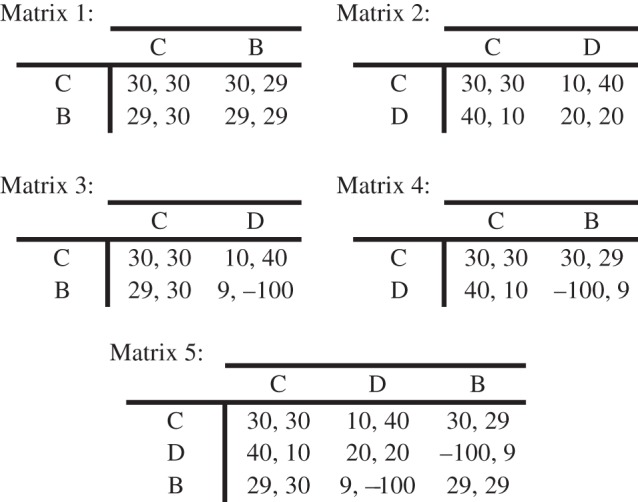
Experimental games consisting of four decomposed matrices (Matrix 1–4) and one full matrix (Matrix 5) corresponding to the Boobytrap game.

The specific pay-off values from the Boobytrap game matrix in figure 2*a* were used for the experiment (as reproduced in figure 3, bottom). To reduce the complexity of the matrix and to isolate participants' preferences for different strategies within different contexts, the 3 × 3 ‘full’ matrix was also decomposed into a set of 2 × 2 matrices with the same pay-off values per included cells, as shown in figure 3. The first 2 × 2 matrix (figure 3, top-left) is a simple symmetric game in which both players choose between C and B strategies. Matrix 2 (figure 3, top-right) is another symmetric game with choices between C and D; this instantiates the standard PD game and provides a baseline rate for participants’ defection without the option of B. Matrix 3 and Matrix 4 (figure 3, centre) each cross the options of C and B for one player against the co-player's options of C and D. Crucially, for Matrices 3 and 4, a virtual bargaining account predicts a mixture of ‘*CC*’ and ‘*CB*’ outcomes (i.e. (C,C), (C,B) and (B,C) equilibria) over games, whereas Nash equilibrium theory predicts the ‘*CD*’ outcome (i.e. (C,D) or (D,C) equilibria; see also discussion below). Therefore, these ‘reduced’ versions of the Boobytrap game capture relevant dimensions of interest in the full Boobytrap version, while also making contrasting predictions from Nash and virtual bargaining perspectives.

Using the z-Tree (Zurich Toolbox for Readymade Economic Experiments) software [31] to present the five experimental matrices over 18 networked computers, participants played the various games during one group session in the same laboratory. Prior to play, participants were instructed on how to ‘read’ game matrices (i.e. understand what rows, columns and cells indicate), and then viewed the games in matrix form on their computer screen. Each participant was randomly and anonymously matched with a new partner for each game, and played in one of two possible roles (Row or Column player) throughout the experiment.

The four 2 × 2 matrices were each presented 20 times, in random order, for a total of 80 games. This was then followed by 10 presentations of the full 3 × 3 matrix. To make the pay-offs easier to read, some minimal text was added within cells (e.g. ‘*Row gets 40*’, ‘*Column gets 10*’). To control for position effects, the locations of strategies in the 2 × 2s were randomized and counterbalanced for each subject, so that on any given trial, each subject saw a different configuration of the same matrix. To avoid label effects, participants did not see strategy labels (e.g. *C*, *D*) for any matrices, but instead clicked on unlabelled buttons alongside each column or row to indicate their selection. Participants made their choice without knowledge of the other player's decision and received feedback on their screen regarding the outcome of their joint decision after each game.

### (b) Results and discussion

**Figure 4.**
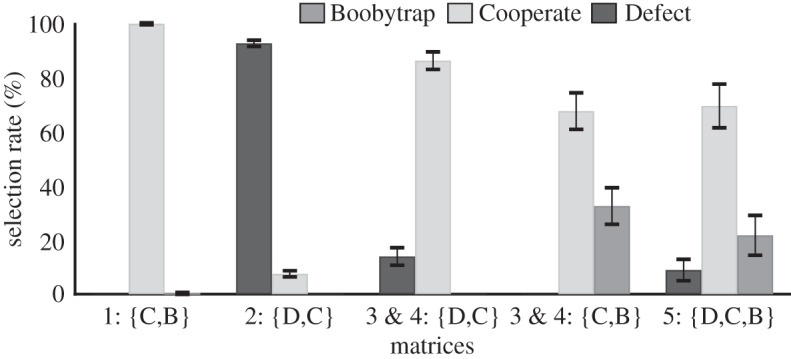
Mean selection rate of strategies B (Boobytrap), C (Cooperate) and D (Defect) across matrices. Choices for matrices 3 and 4 were combined and grouped according to the two strategy sets available to participants: D or C; C or B.

The mean rates for choosing the various strategies per game were calculated for each participant and then averaged to obtain the group means displayed in figure 4. For comparative purposes, because matrices 3 and 4 were asymmetric (each involving different strategy options per role) and largely identical (i.e. with the same strategies, but reversed by role), choice frequencies were combined across these two matrices with respect to participants’ paired options (i.e. C or B; C or D). Specifically, the observed rates at which the Column player chose a given strategy for Matrix 3 (i.e. C or D, in the context of the co-player choosing C or B) were grouped with the rates for which a Row player chose between the same strategies in the same context for Matrix 4 (again, C or D, when the other player may choose C or B). Similarly, Row's choices for Matrix 3 were averaged with Column's analogous choices for Matrix 4 (i.e. C or B, when the other chooses C or D).

As displayed in figure 4, participants overwhelmingly chose the cooperative strategy C (99.7%) in Matrix 1 when confronted with a decision between C or B*.* Because C should clearly be the preferred choice in this context, participants’ selections provide a basic check that they understood how to interpret the abstract matrices. For Matrix 2, participants chose the Defect, over Cooperate, strategy a vast majority of the time (92.5%); thus, without the Boobytrap strategy, the baseline rate of defection for the standard PD game is very high. For the reduced versions of the Boobytrap game in Matrix 3 and 4, this preference reverses; when deciding between C or D in the context of the other's C or B, participants chose Cooperate 86.1% of times. Further, when deciding between C or B in the context of the other's C or D, participants chose Boobytrap about a third of the time (32.5% B, 67.5% C).

To check whether potential learning during the experiment may have influenced results, we recomputed the means displayed in figure 4 for the first and second halves of the experiment (involving the 2 × 2s), as well as for the first and last half of presentations of the 3 × 3 matrix. For the reduced Boobytrap games, a difference across experimental halves occurs for participants’ choices when constrained to choose between C or D: defection decreases from 23% to 7%, with a concomitant increase in cooperation (*t*_17_ = 2.79, *p* = 0.01); but the mean rate of boobytrapping does not differ (*p* = 0.21). There are no significant changes evidenced for the other game matrices, including the full 3 × 3 Boobytrap game. Thus, even with the change noted above, performances in boobytrap games do not conform to standard Nash predictions throughout (more on this below). Importantly, for the boobytrap matrices, defection is low and boobytrapping is present from the onset (i.e. while 72% defection is observed for the first presentation of standard PD, defection is *below* 25% for the first presentation of Matrix 3 and 4, and boobytrapping begins at the rate of 1/9). As none of the individual choice patterns in this section change when considering experimental halves separately, we report subsequent analyses across the whole of the experiment.

Accordingly, the overall distribution of choices in the full, symmetric version of the Boobytrap game (Matrix 5) also shows a high proportion of Cooperate and Boobytrap strategies relative to Defect: 69.4% C, 21.7% B and 8.9% D. Individuals' selection rates for each of these strategies did not differ from their rates for the strategies' counterparts in the reduced Boobytrap games shown in figure 4 (with cooperation rates averaged), matched-pair comparisons: all *p*s > 0.05. This resulted in similar group frequencies to the reduced versions (Matrix 3: 78.6% C, 14.4% B, 6.9% D; Matrix 4: 75% C, 18.1% B, 6.9% D) and indicates that neither the mere availability of a third option as such, nor the added complexity of the matrix in 3 × 3 form are principally driving participants’ results. Yet, there was a significant difference in defection across Boobytrap games (reduced and full versions) and PD games in figure 4 (one-way repeated measures ANOVA: *F*_2,34_ = 199.59, *p* < 0.001), with planned comparisons revealing a dramatic reduction in the proportion of defection from PD to that in the reduced Boobytrap game (when constrained to choices C and D), CI_95_ = 0.66 to 0.92, *p* < .001, and from PD to the full Boobytrap game, CI_95_ = 0.70 to 0.98, *p* < 0.001.

Following §3, virtual bargaining predicts a mixed strategy of ‘cooperative’ choices (C and B) in which B is played sufficiently frequent so as to deter the other player from D. For example, consider Matrix 3. Row should only need to play the Boobytrap strategy with some probability infinitesimally above 1/14 to rationally deter the Column player from Defect. As boobytrapping increases above this frequency, the expected utility to the Column player from choosing D decreases and defection becomes increasingly disadvantageous. The same holds true for Matrix 4, but with roles reversed. And indeed, in our experimental Boobytrap games, participants did choose B at rates well above the threshold of ≈7%. Strategy D was correspondingly chosen the least often, at rates representing more than a 75% decline from those in the standard PD game.

Alternatively, Nash equilibrium theory predicts the *CD* outcome in the reduced boobytrap matrices; C dominates B for the player with these available strategies (i.e. C provides better pay-offs to B irrespective of what the other player chooses), whereas the other player's best reply to their opponent's expected C is D. So when constrained between C and B, a standard Nash account predicts that one always chooses a pure strategy of C; when constrained between D and C, a Nash account predicts that D should always be chosen. However, this is not observed in participants’ choices from figure 4. When deciding C or B, participants did choose C with a high frequency, but B was still observed one-third of the time, significantly more than zero (*t*_17_ = 4.41, *p* < 0.001). And when deciding D or C, participants chose C significantly more than D (*t*_17_ = 9.34, *p* < 0.0001), in direct contrast to the Nash equilibrium prediction.

Table 1 displays the outcomes observed for each game. For PD, participants’ high defection rate results in a mere 1% of outcomes that are mutually cooperative and 86% of outcomes with mutual defection. For the reduced boobytrap matrices, consistent with the above reporting of individual choice, we observe that participants converge on the Nash equilibrium outcome—(C,D) or (D,C)—a minority of times (11 and 8%), whereas the virtual bargaining predicted outcomes ((C,C), (C,B), (B,C)) are the most frequent (ranging from 61 to 26%). Similarly, in the case of the full Boobytrap game, results accord with the virtual bargaining account: combinations of cooperation and boobytrap strategies constitute 82% of game outcomes, with unilateral defection constituting the small remainder. Notably, under these conditions, the Nash equilibrium of (D,D) was never attained.

**Table 1.** Proportion and number of game outcomes per game matrix. PD, Prisoner's Dilemma; BT, Boobytrap game. Column headings represent players' joint outcomes, arising from choice combinations among the strategies C, B and D. Greyed cells indicate unattainable outcomes for a given matrix's strategies.

	(C,C)	(C,B)/(B,C)	(B,B)	(D,B)/(B,D)	(C,D)/(D,C)	(D,D)
Matrix 1	0.99 (179)	0.01 (1)	0.00 (0)	—	—	—
Matrix 2 (PD)	0.01 (1)	—	—	—	0.14 (25)	0.86 (154)
Matrix 3 (2 × 2 BT)	0.61 (109)	0.26 (46)	—	0.03 (6)	0.11 (19)	—
Matrix 4 (2 × 2 BT)	0.56 (100)	0.31 (55)	—	0.06 (10)	0.08 (15)	—
Matrix 5 (3 × 3 BT)	0.48 (43)	0.30 (27)	0.04 (4)	0.04 (4)	0.13 (12)	0.00 (0)

## 5. Alternative frameworks for understanding the boobytrap game

We have focused on the contrast between our virtual bargaining account of the Boobytrap game and the standard Nash equilibrium in the game: (Defect, Defect). But there are, of course, many other models in game theory that might be applied to the Boobytrap game.

One approach is to enrich the agent's utility functions to include ‘social preferences’ [32] by assuming that players attend not just to their own pay-offs, but also to the pay-offs of the other player. This would provide a possible explanation for playing B rather than C, even though the pay-offs to oneself are always greater when playing C. That is, there might be an additional ‘boost’ to one's utility on observing that the other's pay-off is low or, perhaps crucially, substantially lower than one's own.

If this is correct (e.g. if a player prefers to receive ‘29’ while the other receives ‘−100,’ rather than both players receiving ‘30’), then the decision to choose the Boobytrap can also be maintained if the choice of the other player is randomly determined. Specifically, suppose that in the full 3 × 3 Boobytrap game, the co-player's move is determined by flipping a coin, such that the co-player chooses C or D with probability 1/2 and is constrained to not play B. If it is the *outcomes* received by each player that matter, then the process by which those outcomes are achieved should not be important. If the first player sufficiently prefers the (29,−100) outcome to the (30,30) outcome (enough to offset the small cost of ‘buying’ the Boobytrap), then that player will choose B rather than C.

By comparison, though, completely different results are expected from a virtual bargaining viewpoint. A player cannot form a virtual bargain with a second player whose behaviour is being determined by a random device, as the second player is in no position to implement any virtual agreement. Hence, other things being equal, the virtual bargaining account would predict that the frequency of the non-random player deciding to choose B would be substantially reduced under these circumstances. This contrast in predictions thus provides the basis for a strong empirical test between the present virtual bargaining account and accounts based purely on outcomes, which awaits future experimental work.

A second alternative way to explain the choice of B in the Boobytrap game is as a result of ‘noise’ in the choice process. For example, a popular generalization standard Nash equilibrium is so-called Quantal Response Equilibrium (QRE [33]), according to which players do not, at equilibrium, deterministically choose the *best* response (i.e. the response yielding the maximum expected utility), given the probability distribution over the other's moves. Rather, players are more likely to choose a response to the extent that it has a high expected utility. Depending on a free parameter, which determines the noisiness of responding, QRE's predictions range from completely random responding (with each response having a probability of one-third for both players) to collapsing back to the Nash equilibrium as the level of decision noise tends to zero.

Thus, with sufficient noise, QRE can predict B is played fairly frequently—and where it is played frequently enough to successfully deter D, then D will be suppressed, as observed in our experiment. Note, though, that QRE makes the very strong prediction that the frequency of C responses must always be greater than the frequency of B responses. This is because C has a higher pay-off than B whatever the other player does; that is, C dominates B, and frequencies are positive monotonic functions of expected pay-off.

**Figure 5.**
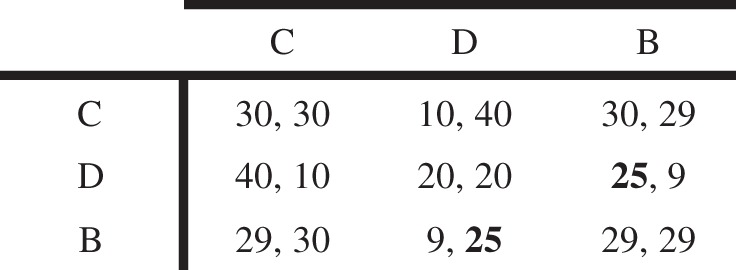
A variation of the Boobytrap game. See text for explanation.

Now consider the Boobytrap game in figure 5. Here, the ‘boobytrap’ only causes a small amount of ‘damage’ to the other player, reducing the defector's pay-off to ‘25’, rather than ‘−100’. Virtual bargaining predicts that B should be played with a high probability and much greater than that of C, under reasonable assumptions. This is because the boobytrap will only have the appropriate ‘deterrent’ effect if it is almost certain to occur, given that the harm it causes is relatively slight.

By contrast, QRE predicts a very different pattern. Because B is constrained to be less frequently chosen than C, it cannot be chosen sufficiently often to be an adequate ‘deterrent’ to the choice of D. So D will be the most frequent choice, followed by C, and finally B. Exploring what happens when the boobytrap is less ‘potent’ is thus a critical test between QRE (and noise-based models more broadly) and the virtual bargaining account, and the natural topic for future experimental work.

## 6. Concluding remarks

In humans, whether we achieve our goals often depends not only on our own actions, but on the actions of others. In such cases, successful goal-directed behaviour often requires two or more people to coordinate their actions successfully, whether in passing each other in the corridor without collision, playing a duet or a game of football, collaborating on a research project or a business venture, or dividing up and carrying out tasks within a family unit. The theory of virtual bargaining aims to provide the starting point for an account of how this is possible.

We have considered virtual bargaining in cases where the goals of the players may be diametrically opposed (as in PD, the starting point for the Boobytrap game, wherein choosing D rather than C is in each player's interests at the expense of the other). But virtual bargaining is also applicable in contexts where players have common goals, but the challenge is to infer how to coordinate their behaviour to achieve those goals. Arguably, such problems of coordination are central to a vast range of human activities, in which people must jointly attend to the same information, jointly plan how to react to that information and jointly engage in appropriate actions—as exemplified in a range of activities including conversation, collaborative musical or theatrical performance, many types of improvised dance and collaborative projects from putting up a tent to writing an academic paper.

As noted earlier, a particularly simple example of such coordination games is the Hi–Lo game, in which players must independently choose to respond H (‘Hi’) or L (‘Lo’). Recall that if the players choose different options, neither obtains any reward. However, if the players both choose Hi, they both receive a large prize, and conversely, if they both choose Lo, they both receive a small prize. Experiments consistently show, as intuition would expect, that Hi–Hi is overwhelmingly chosen [8].

A full evaluation of the virtual bargaining theory would, of course, require a much broader review of the experimental literature on games, and this would go far beyond the scope of this paper. We note, though, that the scope of the present account is quite broad. Consider, for example, the Ultimatum Game. In one version, Player 1 announces a ‘split’ of, say, $100; Player 2 announces a ‘minimum’ sum to which they will agree. If Player 1's split is less than Player 2's minimum, neither player gets anything. Otherwise, the players receive the split as suggested by Player 1. Under fairly natural symmetry assumptions, the best bargain (judged by Nash bargaining) will be a 50–50 split; and virtual bargaining then suggests that this bargain will be implicitly ‘agreed’ by the players. Indeed, the 50–50 split is the modal outcome in many experiments. This provides an alternative, or perhaps complement, to standard economic accounts based on fairness [34]. A natural test between the approaches would be a version of the ultimatum game with three players: Player 1 suggests a split between the three players; Player 2 specifies a minimum payment for both Player 2 and Player 3; Player 3 is a bystander. An account based purely on fairness would presumably require even splits between the three players; a virtual bargaining account might predict a greater share demanded by, and given to, Player 2 rather than Player 3 (data from Güth & Van Damme [35] suggests that fairness may play only a small role).

It is natural to view communication itself as a coordination game of the type above, but where both sender and receiver of a communicative ‘signal’ must coordinate on the same interpretation of that signal, within the space of a vast number of possible interpretations. Facial expressions, gestures and utterances all, notoriously, hugely underspecify the content that is to be communicated. Only by extremely complicated processes of ‘pragmatic enrichment’ is it possible to interpret a raised eyebrow as, perhaps, expressing the implausibility of a specific philosophical position, or to interpret a brief shudder as a request to close a draughty window [36]. Communication involves placing a communicative action of some kind in common ground between ‘sender’ and ‘receiver’; and communication succeeds when, of the endless possible interpretations of the communicative act, both sender and receiver coordinate on the same interpretation. Such communication may be linguistic, or entirely non-verbal, as in conducting an orchestra, where the complex movements of the players should be coordinated by subtle gestures from the conductor [37]. How far convergence on the same interpretation involves explicit inference [38] or is a side-effect of common processing mechanisms between speaker and hearer [39,40] remains a key area for future research.

Note that, in the current framework, communication requires common knowledge that the action to be interpreted is communicative: both sender and receiver aim to infer the *same* interpretation as each other. This contrasts with animal signalling such as ‘stotting’ where a gazelle's jumps in the presence of lions may signal its fitness [41] and hence that the lion should not expend energy in futilely chasing it; or exaggerations of normal human movements (e.g. a goal-keeper conspicuously looking at a defender to indicate where he will pass the ball). In these cases, transfer of information still occurs whether or not the receiver recognizes the intention behind the action—the lion can infer the futility of the chase and the defender can infer the likely direction of the pass from the movements themselves (only fast gazelles can jump; goalies normally kick in the direction they are looking). By contrast, the goalie *pointing* to a defender only gives information about the likely pass if the point is interpreted by the defender as communicative (so the goalie and defender must agree on the likely *referent* of the pointing action), rather than, say, as a random wave of the arm. Empirically clarifying the boundary between cases where signal is *sent intentionally*, which can be very intricate [42], and those which require *common knowledge of the intention of the signal* is an important topic for future work. The present approach can be viewed as a step towards providing a formal underpinning for the theory of psychological communication developed by Clark and co-authors [40,43,44] and in pragmatic theory [36].

We see the foundations of understanding human communication as a particularly rich area for the application of the theory of virtual bargaining. Moreover, if we are right, then virtual bargaining underpins the ability to bargain explicitly, using linguistic or other modes of communication; without virtual bargaining, we suggest, communication, and the complex cultural structures built upon it, would not be possible.

## Supplementary Material

Supplementary material
