# Supplementary material for: Virtual bargaining: a theory of social decision-making
Source: Philos Trans R Soc Lond B Biol Sci. 2014 Nov 5;369(1655):20130487. doi: 10.1098/rstb.2013.0487 (PMC4186239; doi:10.1098/rstb.2013.0487)
Supplement: Supplementary material [file rstb20130487supp1.pdf]

Misyak, JB and Chater, N. 2014. Virtual bargaining: a theory of social decision-making. *Phil. Trans. Roy. Soc. B.* **369** doi: 10.1098/rstb.2013.0487

Supplementary Materials

1. Virtual bargaining with an adversary: The example of "chicken"

Note that the type of case captured by the Boobytrap game is actually very widespread in social behaviour. The threat to engage in conflict, whether explicit or implicit, if exercised by one party in an interaction, typically has bad consequences for both parties. For example, if two animals fight, two people enter a legal dispute, or two armies clash, the outcome may be far worse for both sides than any agreement between them, however asymmetrical. Threats are non-credible under these conditions from the point of view of Nash reasoning: no agent maximising its own payoffs will go through with a threat, so both players can deduce that hostilities will not arise.

Consider, for example, an asymmetrical game of 'chicken' (figure S1) in which both players can 'dare' to Go, or play safe and Stay. Any player who chooses Stay gains the guaranteed, low payoff of one unit. If just one player chooses Go, they gain a good payoff of five units. If they both choose Go, this is a mutually bad outcome—but especially so for the Row player (-50 units). We might imagine, for example, two drivers want to manoeuvre into the same parking space: if they both go for it, the resulting crash is mutually bad. But suppose that one drives a sports car and the other drives a battered SUV. Then, the sports car is liable to suffer by the greater damage.

This game has two pure strategy Nash equilibria of (Go, Stay) and (Stay, Go) (and a mixed strategy, where each player occasionally plays Go, with frequencies small enough that the expected payoff of both players is one unit, whether they Go or Stay). But, we suggest, that in practice, the 'less vulnerable' agent will typically play Go exclusively and the 'more vulnerable' agent will play Stay. The battered SUV will take the parking space. According to the virtual bargaining viewpoint, this results from the common knowledge that, were they bargaining 'for real,' the sports car owner would be less able to credibly 'threaten' to go for the parking space; so the battered SUV can safely proceed unimpeded. Similarly, more powerful agents (whether individuals, companies, or nations) can credibly 'bully' weaker agents by the threat of conflict even if conflict would be negative for both.

This type of case highlights that virtual bargaining is applicable, even where the parties involved are adversaries rather than collaborators. Even if the drivers have a history of animosity, and the sports car driver is outraged by the virtual bargain favouring the other, it is common knowledge to both drivers that the sports car driver cannot unilaterally violate that virtual bargain, without causing disastrous damage to a valuable car.

	go	*stay*
go	-50, -1	5, 1
stay	1, 5	1, 1

Figure S1. Game of chicken, with asymmetrical payoffs.

2. A formal sketch of some aspects of a theory of virtual bargaining.

We can capture the notion of a *feasible bargain*, and contrast this with the narrow notion of a Nash equilibrium as follows. Consider a game with *n* players, where M_i is the set of possible moves for player *i*. An *n*-tuple of moves, one from each player, is denoted $\mathbf{m}=(m_1,\ldots, m_i\ldots m_n)$, where $m_i \in M_i$ and $\mathbf{m} \in \mathbf{M} = M_1 \times \ldots \times M_i \times \ldots \times M_n$. A game, $\mathbf{G}(\mathbf{m})=\mathbf{u}$, assigns a real-valued *payoff* to each player *i*, i.e., it outputs an *n*-tuple $\mathbf{u}=(u_1,\ldots, u_i\ldots u_n)$, as a function of the *moves* of all the players, i.e., the *n*-tuple $\mathbf{m}=(m_1,\ldots, m_i\ldots m_n)$. A *strategy* P_i, for player *i* is a probability distribution over the moves available to that player—i.e., over the elements of M_i. (A *pure* strategy corresponds to a probability distribution in which a single move has probability 1; all other strategies are *mixed* strategies. This distinction will not be important here.) An *n*-tuple of strategies $\mathbf{P}=(P_1,\ldots, P_i\ldots P_n)$ defines a probability distribution over the *n*-tuple of moves, one from each player, $\mathbf{m}=(m_1,\ldots, m_i\ldots m_n)$, such that $\mathbf{P}(\mathbf{m})=\mathbf{P}(m_1,\ldots, m_i\ldots m_n)= \prod_{i=1}^{n} P_i$, that is, each player chooses independently. The *expected payoffs* of each player, given a set of strategies, $\mathbf{P}$ are therefore: $\mathbf{EU}(\mathbf{P}) = \sum_{\mathbf{m}\in\mathbf{M}} \mathbf{P}(\mathbf{m})\,\mathbf{G}(\mathbf{m})$. We write the expected payoff for player *j* to be the *j*th component of this n-tuple: $\mathbf{EU}_j(\mathbf{P})$.

Let us write $\mathbf{P}(P_i \to Q_i)$ for the set of strategies $(P_1,\ldots, Q_i\ldots,P_n)$, i.e., where player *i*'s strategy P_i has been substituted with a different strategy Q_i, where both P_i and Q_i are defined over player *i*'s possible moves M_i.

An *n*-tuple of strategies $\mathbf{P}=(P_1,\ldots,P_n)$ is a Nash equilibrium just when

$$\nexists i[\exists Q_i[\mathbf{EU}_i\big(\mathbf{P}(P_i \to Q_i)\big) > \mathbf{EU}_i(\mathbf{P})]]$$

That is, there is no person *i* who can switch from strategy *Pi* to *Qi* to strictly improve their expected payoff.

An *n*-tuple of strategies $\mathbf{P}=(P_1,\ldots,P_n)$ is a *feasible* bargain just when

$$\nexists i[\exists Q_i[[\mathbf{EU}_i\big(\mathbf{P}(P_i \to Q_i)\big) > \mathbf{EU}_i(\mathbf{P})]\&[\exists j \mathbf{EU}_j\big(\mathbf{P}(P_i \to Q_i)\big) < \mathbf{EU}_i(\mathbf{P})]]]$$

That is, there is no person *i* who can switch from strategy P_i to Q_i to strictly improve their expected payoff while simultaneously strictly reducing the expected payoff of at least one other player *j*. Thus,

all Nash equilibria must be feasible bargains (because if all *i* fail to satisfy the first conjunct, they must clearly fail to satisfy the conjunction).

Now, we can also formally specify a goodness measure over bargains, following the Nash bargaining solution (although accounts of bargaining could be employed to create distinct versions of the virtual bargaining account).

Consider an *n*-person game **G**. Suppose that there is a 'status quo' expected payoff, if the players do not engage in virtual bargaining $\mathbf{EU}^{\boldsymbol{D}} = (\mathbf{EU}_{\mathbf{1}}^{\boldsymbol{D}}, \mathbf{EU}_{\mathbf{2}}^{\boldsymbol{D}}, \dots, \mathbf{EU}_{\boldsymbol{n}}^{\boldsymbol{D}})$ (The principles for determining, in general, a default value for an arbitrary game is a topic for future research. In Nash's bargaining account, a status quo payoff, where the players do not reach a bargain is assumed.). The benefit of a bargain in which the players choose strategies **P**=(P_1, P_2,…, P_n) for player *i* is $\mathbf{EU}_{\boldsymbol{i}}(\mathbf{P}) - \mathbf{EU}_{\boldsymbol{i}}^{\boldsymbol{D}}$ (here, we are conceiving of benefits in terms of 'utility' rather than, say, monetary payoffs; for small amounts of money, from a normative point of view, utility should be nearly linear in money [1], so that this difference is not critical). The goodness of a bargain can be captured by the 'Nash product' of the utility gained by each player: $\prod_{\boldsymbol{i}=\mathbf{1}}^{\boldsymbol{n}}(\mathbf{EU}_{\boldsymbol{i}}(\mathbf{P}) - \mathbf{EU}_{\boldsymbol{i}}^{\boldsymbol{D}})$.

A final step in the theory of virtual bargaining is to determine which bargain is chosen. The obvious criterion is to choose the bargain with the highest goodness rating, as in the Nash solution. Where there is a clearly 'best' bargain, then this choice is typically also appropriate for *virtual* bargaining. But where, for example, there are severally equally 'good' bargains, then the virtual bargaining case is more complex; given that the bargaining process is entirely virtual, so that no information is exchanged between players, it may not be straightforward to choose from among a set of equally good bargains. Interesting situations of this sort, with relevant empirical results, have been described [2]. We leave the exposition of an account of choice in virtual bargaining for later work.

References

[1] Rabin, M. 2000 Risk aversion and expected-utility theory: A calibration theorem. *Econometrica* **68**, 1281-1292. (doi:10.1111/1468-0262.00158)

[2] Bardsley, N., Mehta, J., Starmer, C. & Sugden, R. 2010 Explaining focal points: Cognitive hierarchy theory versus team reasoning. *Econ. J.* **120**, 40-79. (doi:10.1111/j.1468-0297.2009.02304.x)
